# Comparative analysis of the gut microbiome of ungulate species from Qinghai–Xizang plateau

**DOI:** 10.1002/ece3.70251

**Published:** 2024-09-10

**Authors:** Xibao Wang, Xiaodong Gao, Yao Chen, Xiaoyang Wu, Yongquan Shang, Zhihao Zhang, Shengyang Zhou, Honghai Zhang

**Affiliations:** ^1^ College of Life Sciences Qufu Normal University Qufu Shandong China

**Keywords:** gut microbiome, niche, phylogeny, wild ungulates

## Abstract

Several studies have investigated the gut bacterial composition of wild ungulates in the Qinghai–Xizang Plateau. However, the relationship between their gut microbiome dendrograms and their phylogenetic tree remains unclear. In this study, we analyzed 45 amplicons (V3–V4 region of the 16S rRNA gene) from five wild ungulates—*Pseudois nayaur*, *Pantholops hodgsonii*, *Gazella subgutturosa*, *Bos grunniens*, and *Equus kiang*—from the Qinghai–Xizang Plateau to clarify the relationship between their phylogenies and gut microbiome dendrograms. The unweighted pair group method with arithmetic mean analysis and hierarchical clustering analysis indicated that *G. subgutturosa* is closely related to *P. nayaur*; however, these results were inconsistent with their phylogenetic trees. Additionally, the indicator genera in the microbiome of each wild ungulate showed strong associations with the diets and habitats of their host. Thus, diet and space niche differentiation may primarily account for the differences between the gut microbiome characteristics of these wild ungulates and their phylogeny. In summary, our research provides insights into the evolutionary factors influencing the gut microbiome of wild ungulates in the Qinghai–Xizang Plateau.

## Graphical abstract

This study investigated the relationship between the gut bacterial community and the phylogeny of five wild ungulates (*Pseudois nayaur*, *Pantholops hodgsonii*, *Gazella subgutturosa*, *Bos mutus*, and *Equus kiang*) inhabiting the Qinghai‐Tibetan Plateau using 16S rRNA gene data. The results of UPGMA and HCA were inconsistent with those of IQ‐TREE, indicating that host phylogeny is not the only factor influencing the gut microbiota, and other factors, such as diet and habitat, may also have an impact. There were significant differences in gut bacterial composition among the five wild ungulates, and each species had distinctive bacterial features, primarily associated with fiber and cellulose breakdown, contributing to gastrointestinal homeostasis.

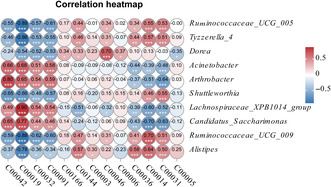

## 1 INTRODUCTION

Vertebrates harbor vast and complex communities of microorganisms in their gastrointestinal tract, including archaea, bacteria, fungi, protozoa, and viruses. Among these, bacteria constitute the highest proportion of these microorganisms (Suchodolski, 2011; Wang, Shang, Wei, Wu, et al., 2022; Xu et al., 2015; Zhu et al., 2022). Several studies on the bacterial communities in vertebrate guts have shown that these microorganisms are crucial in the overall health and well‐being of their host (Muñoz et al., 2024; Spencer et al., 2021; Wang et al., 2019; Xu et al., 2023). The bacterial community maintains gut homeostasis (Singh, 2019; Zmora et al., 2019), digests nutrients (Greene et al., 2020; Shang et al., 2023; Wu et al., 2016), and provides energy (Tremaroli & Bäckhed, 2012; Zhang et al., 2016). For example, the bacterial community in giant pandas can absorb and convert 70% of the flavonoid monomers present in bamboo leaves and shoots (Wang et al., 2021).

The gut microbiome is influenced by various factors such as diet (Brunetti et al., 2023; Huang et al., 2021; Yao et al., 2021; Yarlagadda et al., 2021), temperature (Bo et al., 2019; Koziol et al., 2023; Sepulveda & Moeller, 2020), niche (Sadeghi et al., 2023; Shankregowda et al., 2023; Wang, Shang, Wei, Wu, et al., 2022; Wang, Wu, Shang, Mei, et al., 2022), and phylogeny (Cortes‐Ortiz & Amato, 2021; Hird et al., 2015; Laviad‐Shitrit et al., 2019). Additionally, a previous study indicated that the correlation between gut microbiome and host phylogeny is stronger in mammals than that in birds, reptiles, or amphibians (Song et al., 2020). For example, Brown et al. (2023) observed topological congruence between the phylogenies and microbiome dendrograms of 14 sympatric small mammal species at or above the family level. Conversely, some studies have shown that below the family level, the phylogenies of the subfamilies Cervinae and Caprinae are inconsistent with their gut microbiome dendrograms (Gregor et al., 2022; Li et al., 2018; Sun et al., 2021). Similarly, at the species level, Grond et al. (2020) observed inconsistencies between the gut microbiome dendrograms and phylogenies of six chipmunk species from the western United States. This can be attributed to species with close phylogenetic relationships exhibiting differences in diet and spatial niches to avoid competition. Therefore, at finer phylogenetic scales, spatial and diet niche differentiation among host species can explain inconsistencies between gut microbiome dendrograms and host phylogenies of different species (Brown et al., 2023; Greene et al., 2020).

Previous studies have provided a wealth of data on the 16S rRNA genes of wild ungulates from the Qinghai‐Xizang Plateau (Fu et al., 2021; Liu et al., 2021; Ma et al., 2019; Wang, Wu, Shang, Gao, et al., 2022) that can be used to enhance our understanding of the relationship between their phylogeny and gut microbiome. Therefore, we tested this relationship using 16S rRNA genes. The findings of this study may explain the evolutionary factors affecting the gut microbiome of wild ungulates from the Qinghai‐Xizang Plateau.

## 2 MATERIALS AND METHODS

### 2.1 The 16S rRNA gene data selection

Given the significant differences in captive environments across different regions, we chose wild ungulates to avoid the influence of captivity on the results of our analyses. We used two criteria for selecting data: (1) the sampling period was summer (May–June), and (2) the habitat of the sampled species was the Qinghai‐Xizang Plateau. The sampling environment and 16S rRNA gene data for the five wild ungulates (*E. kiang*, *B. grunniens*, *P. hodgsonii*, *P. nayaur*, and *G. subgutturosa*) were obtained from relevant articles (Qin et al., 2020; Wang, Wu, Shang, Gao, et al., 2022). These wild ungulates belong to two orders, four subfamilies, and five genera. The 16S rRNA gene datasets for *E. kiang* (10 samples named EK), *B. grunniens* (7 samples named BG), *P. hodgsonii* (4 samples named PH), *P. nayaur* (4 samples named PN), and *G. subgutturosa* (20 samples named GS) were downloaded from the SRA database of the National Center for Biotechnology Information (NCBI; www.ncbi.nlm.nih.gov) (Qin et al., 2022; Wang, Wu, Shang, Gao, et al., 2022). Detailed information for the 45 samples is listed in Table S1.

### 2.2 Phylogenetic reconstructions

The mitochondrial genome of wild ungulates, characterized by structural conservation and maternal heredity, is extensively used for identifying phylogenetic relationships among organisms. To reconstruct the phylogenetic relationships of the five wild ungulates, we downloaded the 12 mitochondrial protein‐coding genes (excluding the *ND6* gene) of the five species from the Nucleotide database of NCBI (www.ncbi.nlm.nih.gov). We used MEGA7 software (Kumar et al., 2016) to align the 12 genes. To select the optimal model for the IQ‐tree, we used the model‐defined modules in PhyloSuite software (V1.2.3) (Zhang, Gao, et al., 2020). The IQ‐tree settings were as follows: the optimal model was TIM2; bootstrap ultrafast; bootstrap number was 200,000; maximum number of iterations (max_iter) was 1000; and the minimum correlation coefficient (min_cor_coef) was 0.9. The IQ‐tree was visualized using the Interactive Tree of Life (ITOL) website (https://itol.embl.de/) (Letunic & Bork, 2021).

### 2.3 Sequence processing and analyses

The sequencing region of all samples was the V3–V4 high‐variance region of the bacterial 16S rRNA gene, and all were paired‐end reads using the Illumina platform (San Diego, CA, USA). We used EasyAmplicon software (V1.18.1; Liu et al., 2023) to merge the 45 samples and remove primers. Sequence denoising (Callahan et al., 2017) and chimera removal (Edgar et al., 2011) were performed using the Parallel‐Meta Suite (PMS, V3.7; Chen, Li, et al., 2022). Following the 97% conventional sequence criterion, we used the vsearch tool (Rognes et al., 2016) to obtain the operational taxonomic unit (OTU) table for each sample. Based on the OTU tables and the SILVA database (V123), we obtained each taxon's relative abundance table from the kingdom to species level. At the OTU level, rarefaction curves, species accumulation boxplots, alpha analyses, and analysis of similarities (ANOSIM) were generated using the Tutools platform (http://www.cloudtutu.com). Hierarchical clustering analysis (HCA) and unweighted pair group method with arithmetic mean analysis (UPGMA) were performed using OmicShare (https://www.omicshare.com/tools) and SRplot (http://www.bioinformatics.com.cn/SRplot), respectively. Additionally, we compared the structures of the graphs obtained from UPGMA, HCA, and IQ‐tree testing to determine the relationships between the phylogeny of wild ungulates and their gut microbiome. At the phylum and genus levels, we used the ggplot2 package in R software (V4.2.1) to generate relative abundance column cumulative plots of the top 10 bacteria. Furthermore, to select the indicator genus with the highest contribution to interspecies variation, we performed random forest analysis using the Wekemo Bioincloud platform (https://www.bioincloud.tech).

### 2.4 Metabolite prediction

Based on OTU tables and sequences, we used the microbiome metabolome integration platform (MMIP; http://bioinfo.jisiasr.org/mmip/index.html) (Gautam et al., 2023) to predict the gut microbiome metabolites of the 45 samples (Module‐I). Detailed information on the metabolites is listed in Table S2. To clarify the role of indicator genera, we used OmicShare tools (https://www.omicshare.com/tools) to explore the correlation between indicator genera and gut metabolites using Spearman correlation analysis (threshold: *R* ≥ 05, *p* ≤ .001).

## 3 RESULTS

### 3.1 Alpha diversity

**FIGURE 1:**
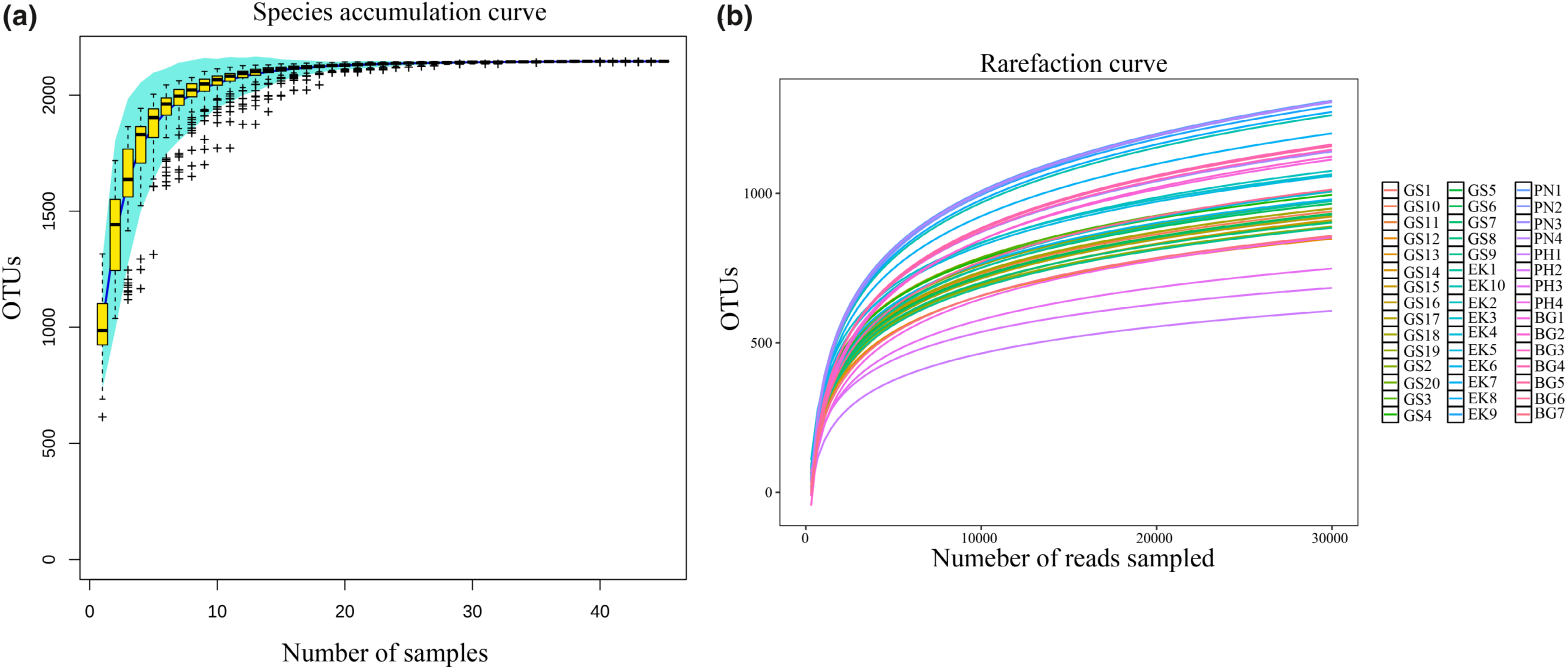
Species accumulation (a) and rarefaction (b) curves. The curves reached a plateau, indicating that the experimental samples were suitable for subsequent analyses.

**FIGURE 2:**
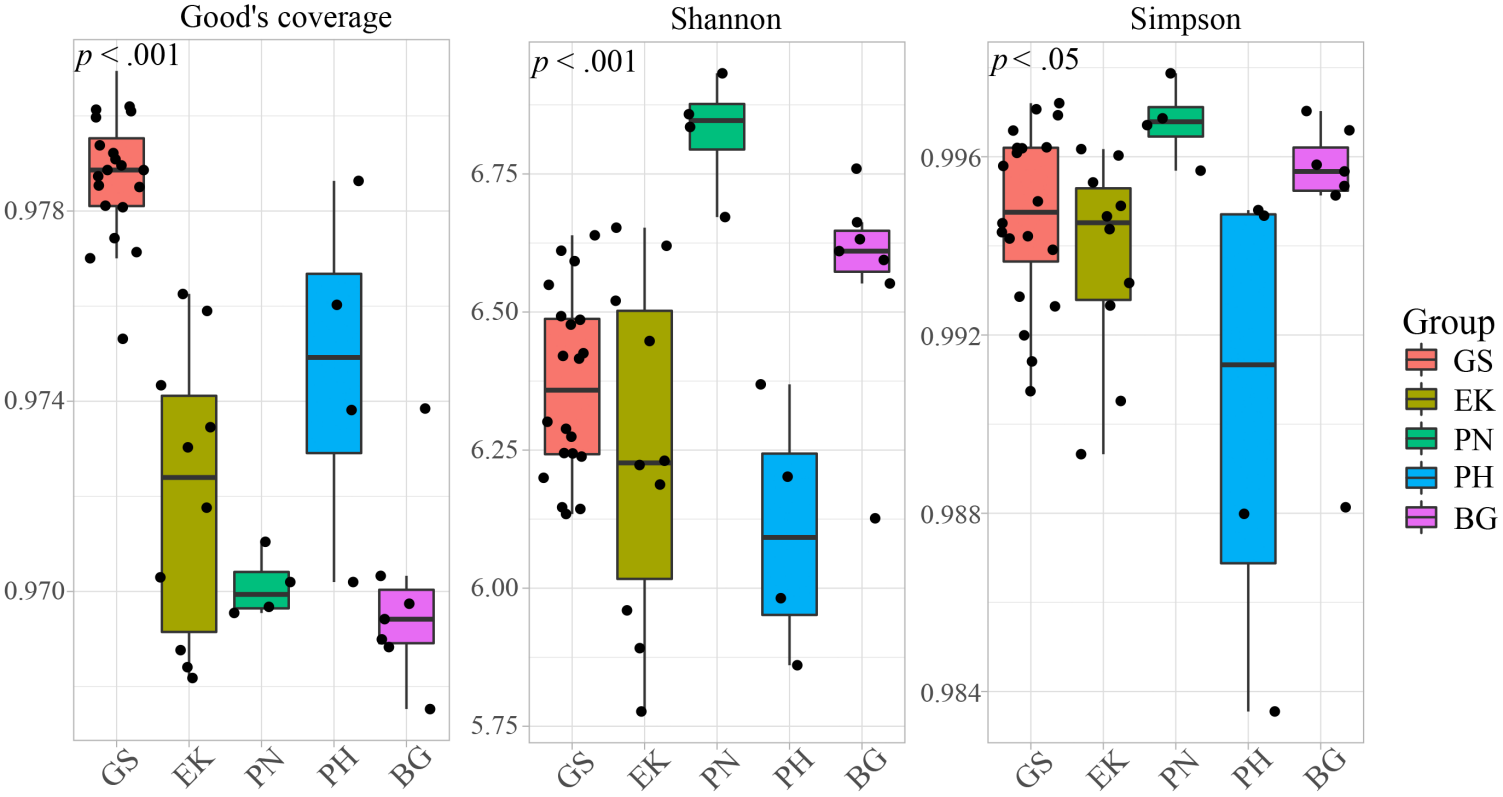
Boxplot of alpha diversity (Kruskal–Wallis test) among the five wild ungulates. *p* < .05 and *p* < .001 indicate significant and extremely significant differences between species, respectively.

The total number of OTUs did not increase significantly when the number of samples exceeded 40 (Figure 1a). The 45 rarefaction curves of the samples gradually flattened at more than 10,000 sampled sequences (Figure 1b). These results indicate that the number of samples and sequencing depth were sufficient for subsequent analyses. All the samples had Good's coverage indices above 0.97 (Figure 2), indicating that our analyses effectively represented the gut microbiome of the five wild ungulates. Our results also showed significantly higher (*p* < .05) Shannon and Simpson indices for PN compared to those of other species. Additionally, the alpha diversity indices of PH were the lowest among the five species (Figure 2).

### 3.2 Gut microbiome composition

**FIGURE 3:**
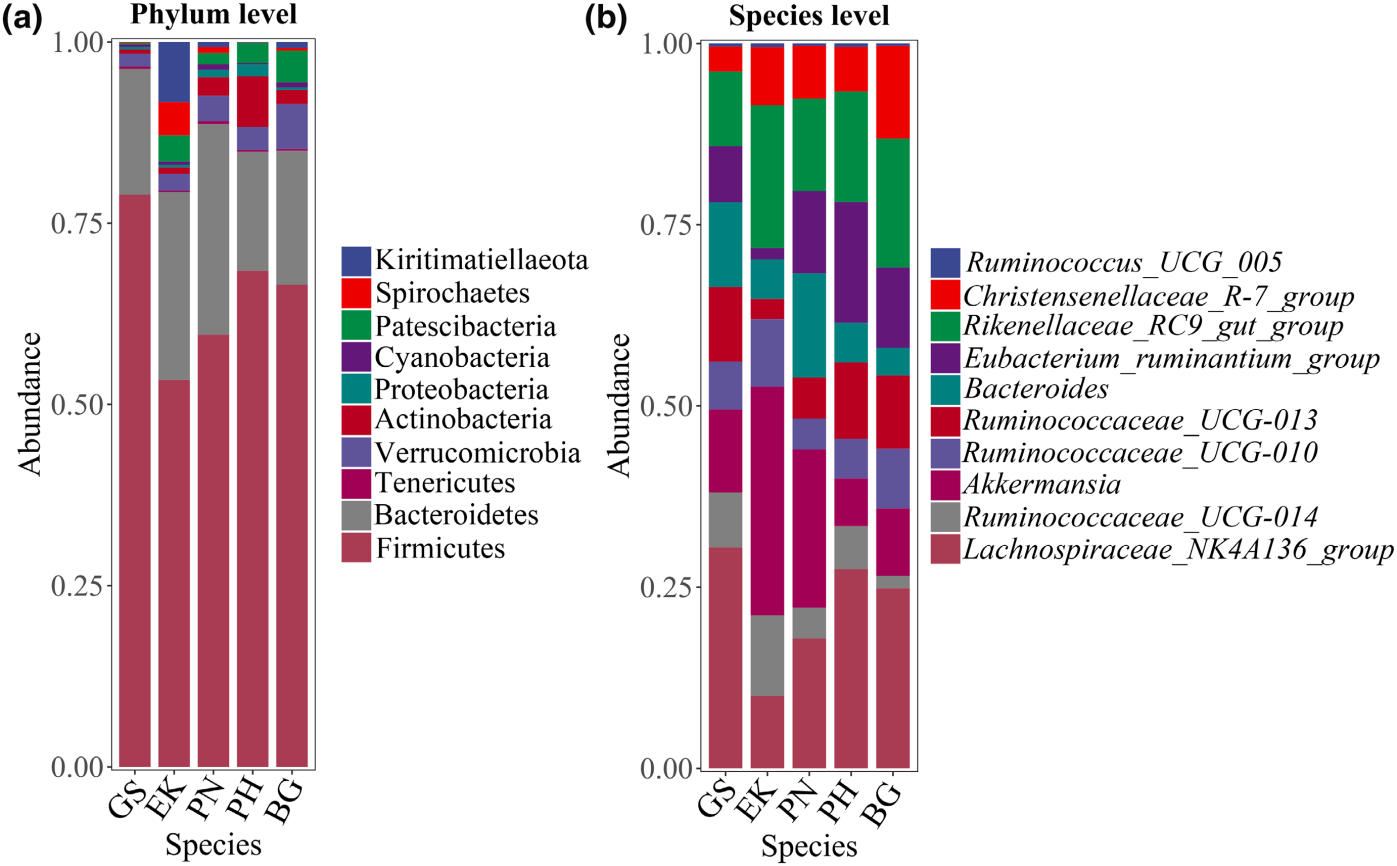
Relative abundances of the top 10 abundant bacteria in the gut of ungulate species at the phylum (a) and genus (b) levels.

At the phylum level (Figure 3a), Firmicutes was the most abundant phylum in the gut microbiome of the five wild ungulates (GS, 77.97%; EK, 53.83%; PN, 58.25%; PH, 68.36%; BG, 66.46%), followed by Bacteroidetes (GS, 18.36%; EK, 25.42%; PN, 30.37%; PH, 17.77%; BG 19.09%). The Firmicutes/Bacteroidetes (F/B) ratio was the highest in GS and lowest in PN (GS, 4.24; EK, 2.12; PN, 1.92; PH, 3.85; BG, 3.48). At the genus level (Figure 3b), *Ruminococcaceae_UCG‐005* was the most abundant genus in four ungulates (GS, 17.80%; PN, 10.19%; PH, 16.87%; BG, 14.48%), except in EK, where the most abundant genus was *Rikenellaceae_RC9_gut_group* (12.44%).

### 3.3 Phylogenetic analysis and cluster analyses

**FIGURE 4:**
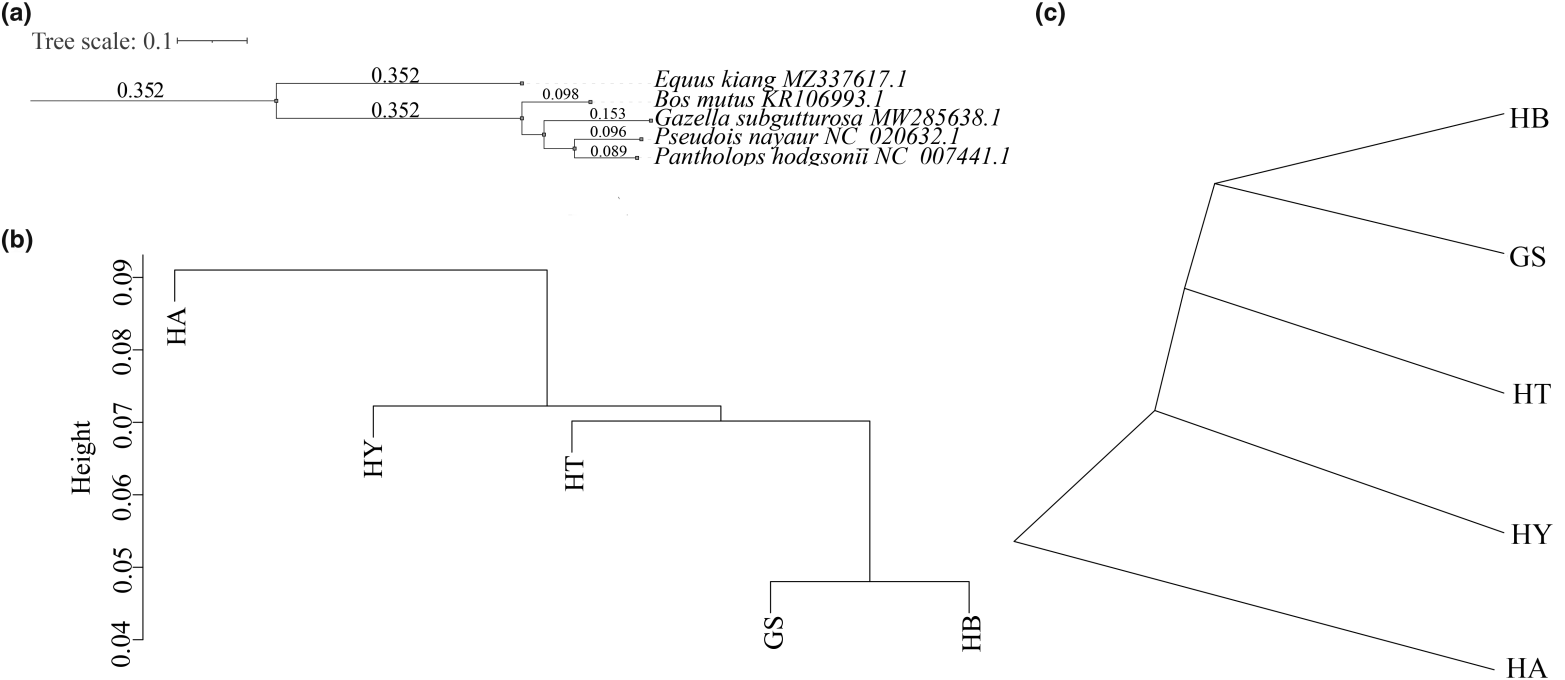
(a) Phylogenetic tree analysis (IQ‐tree; MZ337617.1, KR106993.1, MW285638.1, NC_020632.1, and NC_007441.1 are the mitogenome numbers of the five wild ungulates), (b) hierarchical cluster analysis (HCA), and (c) unweighted pair‐group method with arithmetic mean analysis (UPGMA).

The IQ‐tree showed that *P. nayaur* (PN) and *P. hodgsonii* (PH) clustered into one branch, whereas *E. kiang* (EK) formed the outermost branch (Figure 4a). At the OTU level, HCA and UPGMA showed that GS and PN clustered into one branch, while EK formed the outermost branch (Figure 4b,c). Therefore, the gut microbiome dendrograms of the five ungulates were inconsistent with their phylogenies.

### 3.4 Discrepancies in gut microbiome between species

**FIGURE 5:**
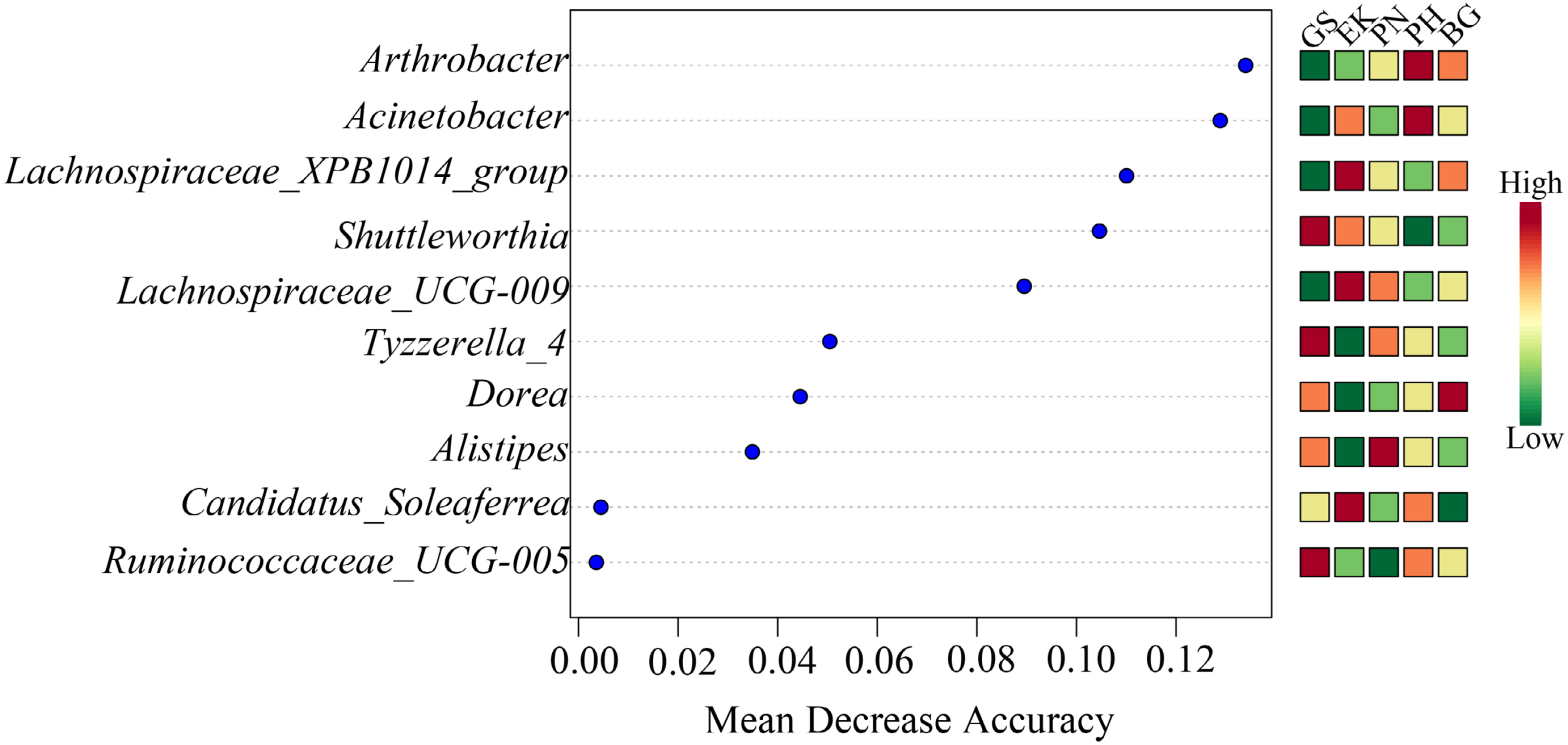
Random forest analysis at the genus level between species. The red squares represent indicator genera.

ANOSIM demonstrated significant differences in gut bacterial composition among the five species (*R* = 0.993, *p* = .001). Consequently, we performed random forest analysis to select indicator genera for the five wild ungulates. At the genus level, *Shuttleworthia*, *Ruminococcaceae_UCG‐005*, and *Tyzzerella_4* were identified as the main bacterial features of GS. For EK, the main bacterial features were *Candidatus_Soleaferrea*, *Lachnospiraceae_UCG‐009*, and *Lachnospiraceae_XPB1014_group*. For PH, they were *Acinetobacter* and *Arthrobacter*, while for PN and BG, they were *Alistipes* and *Dorea*, respectively (Figure 5).

### 3.5 Relationship between the indicator genus and metabolites

**FIGURE 6:**
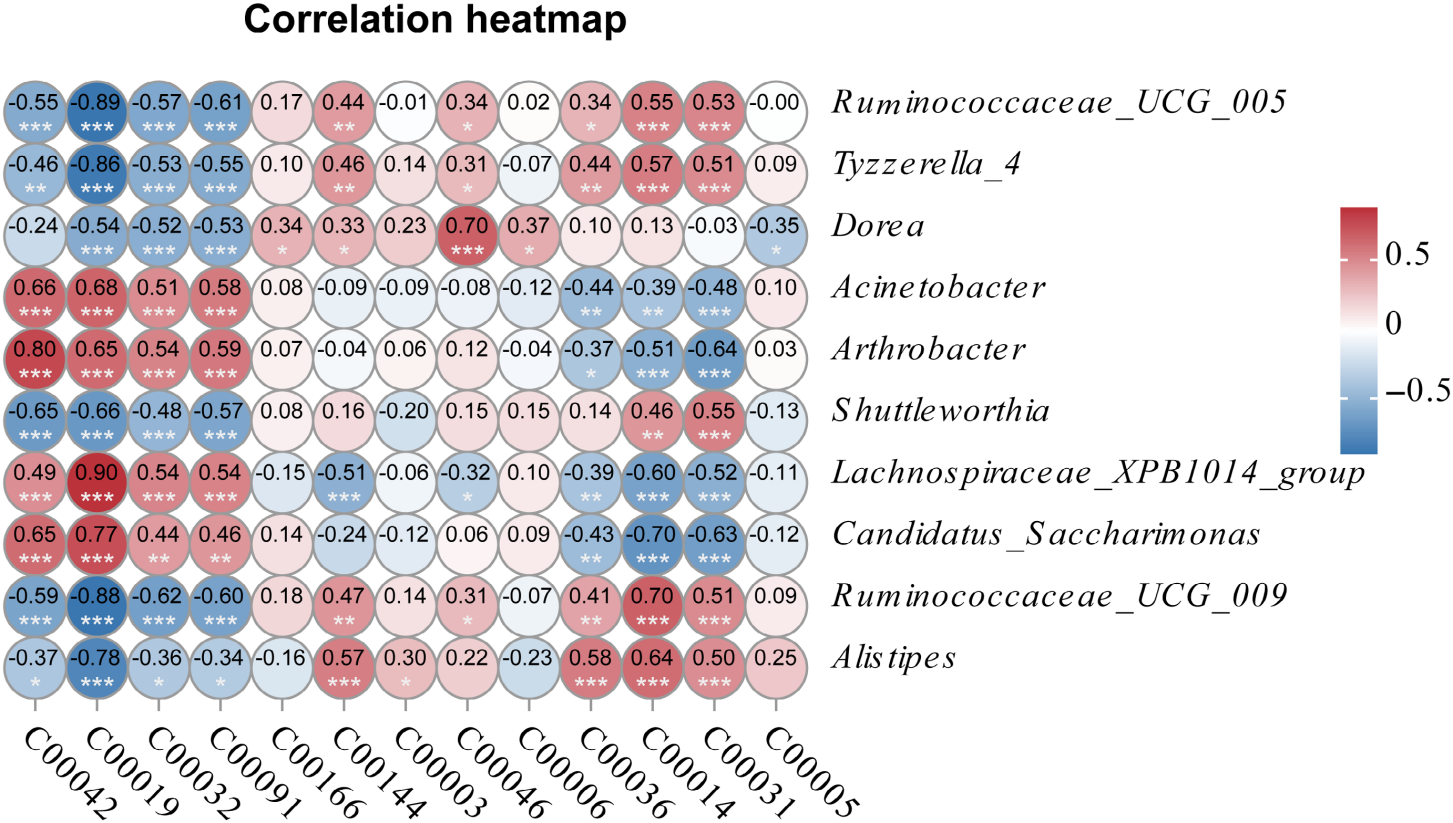
Spearman correlation heatmap showing correlations between indicator genera and metabolites. *p < .05, **p < .01, and ***p < .001. *R* > 0 and *R* < 0 indicate positive and negative correlations between indicator genera and metabolites, respectively.

*Ruminococcaceae_UCG‐005* and *Tyzzerella_4* showed strong and significantly positive correlations with C00031 (D‐Glucose) and C00014 (Ammonia), while *Shuttleworthia* showed a strong and significantly positive correlation with C00031 (*R* > 0.5, *p* < .001, Figure 6). *Lachnospiraceae_XPB1014_group*, an indicator genus in EK, showed strong and significantly positive correlations with C00019 (S‐Adenosyl‐L‐methionine), C00032 (Protoheme), and C00091 (Succinyl‐CoA). *Candidatus_Soleaferrea*, another indicator genus in EK, also showed a strong and significantly positive correlation with C00019 (*R* > 0.5, *p* < .001, Figure 6). *Acinetobacter* and *Arthrobacter*, indicator genera in PH, showed very strong and significantly positive correlations with C00042 (Succinate), C00032, C00019, and C00091 (*R* > 0.5, *p* < .001, Figure 6). Additionally, *Alistipes*, an indicator genus in PN, showed strong and significantly positive correlations with four metabolites: C00144 (Guanosine 5′‐phosphate), C00036 (Oxaloacetate), C00031, and C00014 (*R* ≥ 0.5, *p* < .001, Figure 6). The indicator genus of BG, *Dorea*, only showed a strong and significantly positive correlation (*R* = 0.7, *p* < .001) with C00046 (Ribonucleic acid, Figure 6).

## 4 DISCUSSION

Based on the 16S rRNA gene data from the NCBI database, we investigated the gut bacterial community of five wild ungulates from Qinghai Province, China. We established the phylogenetic relationships among these ungulates based on their gut bacterial communities and mitochondrial genes. The results of cluster analyses were inconsistent with those of the IQ‐tree. Our findings were similar to those previously reported for captive family Cervidae and subfamily Caprinae (Li et al., 2018; Sun et al., 2021). Above the family level, researchers observed a good correlation between gut microbiome dendrograms and host phylogenies (Ley et al., 2008; Rojas et al., 2021). At or below the genus level, the gut microbiome dendrograms of the species were inconsistent with their respective phylogenies (Liu et al., 2019; Sanders et al., 2014). Sun et al. (2021) observed that the gut microbiome dendrograms of *P. nayaur*, *Hemitragus jemlahicus*, and *Ovis orientalis* in the same captive environment were inconsistent with their phylogenetic structures. It is reported that the differentiation of spatial and dietary ecological niches is primarily responsible for ensuring the survival of species with close phylogenetic relationships (Schoener, 1974). During summer, succulent forbs, such as *Allium polyrhizum*, *Zygophyllum rosovii*, and *Astragalus*, constitute the main components of the diet of *G. subgutturosa* (Xu et al., 2012). *Carex* spp. and *Kobresia* spp. have been identified as the dominant components of the summer diet of *B. grunniens*. Graminoids (*Calamagrostis* spp., *Poa* spp., and *Stipa* spp.) and forbs (*Oxytropis* spp.) dominate the summer diet of *P. hodgsoni* and *E. kiang*, respectively (Shi et al., 2016). *Stipa orientalis*, *Elymus longearistatus*, and *Festuca olgae* are the main components of the summer diet of *P. nayaur* (Mishra et al., 2004). These findings highlight the differentiation of dietary ecological niches among wild ungulates. Therefore, niche differences may be one of the factors responsible for the observed inconsistency between gut microbiome dendrograms and host phylogenies in wild ungulates.

In this study, the Shannon and Simpson indices of *P. nayaur* (PN) were significantly higher (*p* < .05) than those of *B. grunniens* (BG), *P. hodgsoni* (PH), *E. kiang* (EK), and *G. subgutturosa* (GS). A higher gut bacteria alpha diversity is associated with a more complex and stable host gut ecology, which is beneficial for overall host health (Chen et al., 2021; Jiang et al., 2021; Stoffel et al., 2020). Unlike the other four species, *P. nayaur* can climb and forage on rock faces. The high alpha diversity of *P. nayaur* indicates a high resistance and adaptability to external disturbances relative to the other species. *Alistipes* showed a strong and significantly positive correlation with oxaloacetate and D‐glucose. Oxaloacetate is a precursor for generating aspartic (Doctor & Oro', 1972) and citric acid (Comte et al., 1997); aspartic acid is positively associated with intramuscular fat content (Chen, Sun, et al., 2022) and participates in host glycolysis regulation via nicotinate and nicotinamide metabolism (Hwang & Song, 2017). Pyruvate, generated from glucose via glycolysis, participates in the citric acid cycle and gradually generates energy substances such as ATP (adenosine triphosphate) and citric acid (Dashty, 2013). *Alistipes* can help *P. nayaur* obtain energy from food to adapt to its unique spatial niches.

In this study, Firmicutes and Bacteroidetes were identified as the two most abundant phyla in the gut bacterial communities of the ungulates, together accounting for more than 78% of the gut bacterial composition. This observation is consistent with previous studies (Bai et al., 2018; Wang, Wu, Shang, Gao, et al., 2022; Zhang, Chen, et al., 2020). Furthermore, it has been reported that Firmicutes and Bacteroidetes decompose cellulose and carbohydrates in food, respectively (Bai et al., 2021; Bird et al., 2019; Zhao et al., 2018). Several studies have indicated that a high abundance of Firmicutes and a high F/B ratio are beneficial for efficient cellulose and fiber breakdown and energy harvesting in wild ungulates (Lan et al., 2017; Wang, Wu, Shang, Gao, et al., 2022; Zhang et al., 2016). S*ubgutturosa*, *Shuttleworthia*, *Ruminococcaceae_UCG‐005*, and *Tyzzerella_4* showed strong and significantly positive correlations with D‐glucose. The result indicated that four genera can digest fiber to generate D‐glucose (Hara et al., 2018; Zhang et al., 2017). D‐glucose is an important energy substance that is easily absorbed by intestinal epithelial cells (Thorens, 1996). Thus, *G. subgutturosa* has a greater ability to break down cellulose and fiber in its diet to absorb and store energy.

*Acinetobacter* and *Arthrobacter*, indicator genera of *P. hodgsoni*, showed strong and significantly positive correlations with succinate, succinyl‐CoA, and protoheme. Succinate and succinyl‐CoA participate in the tricarboxylic acid cycle, producing large amounts of ATP, which enables the host to survive on the plateau (MacLean et al., 2023). Hypoxia can cause a significant decrease in protoheme synthesis (Edwards et al., 2014). In this study, we observed that *Acinetobacter* and *Arthrobacter*, which can produce sufficient amounts of protoheme to help their host adapt to hypoxic environments, were significantly enriched in the gut of *P. hodgsoni*.

*Oxytropis* spp. is the main component of the summer diet of *E. kiang* (Shi et al., 2016). Furthermore, swainsonine, a secondary alkaloid compound in *Oxytropis* spp., induces hepatic inflammation by altering bile acid metabolism in mice (Fu et al., 2023; Lu et al., 2014). Ren et al. (2023) observed that diet supplementation with swainsonine significantly increases the relative abundance of species belonging to the family Lachnospiraceae in the gut of captive pikas. Members of this family are involved in secondary bile acid biotransformation to regulate swainsonine‐induced hepatic inflammation. *Lachnospiraceae_XPB1014_group*, belonging to the family Lachnospiraceae, are known for their cellulolytic capacity (Biddle et al., 2013; Schwarz., 2001). In this study, *Lachnospiraceae_XPB1014_group*, an indicator genus in *E. kiang*, also showed strong and significantly positive correlations with protoheme and succinyl‐CoA contents. Thus, *Lachnospiraceae_XPB1014_group* can cope with cellulose and swainsonine present in plants belonging to the genus *Oxytropis* spp., helping *E. kiang* obtain energy and maintain a healthy state.

In summer, resistant starch is one of the main nutritional components in the diet of *B. grunniens* (Guo et al., 2024). It has been shown that mice on a resistant starch‐rich diet show increased proportions of Dorea and ‘heavy’ RNA in their guts (Herrmann et al., 2018; Taras et al., 2002). In this study, the indicator genus of *B. grunniens*, *Dorea*, showed a strong and significantly positive correlation with ribonucleic acid. Therefore, this indicator genus can help *B. grunniens* break down resistant starch and generate short‐chain fatty acids (Champ, 2004; Herrmann et al., 2018). Thus, *Dorea*, associated with short‐chain fatty acids, is beneficial for *B. grunniens* to obtain energy and maintain gut homeostatic balance (Byrne et al., 2015; den Besten et al., 2013; Xu et al., 2020).

The results of this study showed an association between the indicator genera of the different wild ungulate species and their distinct diets and nutrient compositions. Therefore, niche differences may be one of the factors responsible for the inconsistency between gut microbiome dendrograms and wild ungulate phylogeny. However, 16S rRNA gene sequencing has some limitations. For example, it does not effectively elucidate the functions of indicator genera. Future studies should perform dietary (nutritional) niche association analyses to explain the inconsistency between gut microbiome dendrograms and host phylogeny for wild ungulates. Furthermore, the species investigated in this study are generally endemic to the Qinghai–Xizang Plateau. Therefore, our results may not be applicable to ungulates in other ecological environments.

## AUTHOR CONTRIBUTIONS

**Xibao Wang:** Data curation (equal); formal analysis (equal); project administration (equal); writing – original draft (equal). **Xiaodong Gao:** Formal analysis (equal). **Yao Chen:** Project administration (equal). **Xiaoyang Wu:** Formal analysis (equal). **Yongquan Shang:** Formal analysis (equal). **Zhihao Zhang:** Formal analysis (equal). **Shengyang Zhou:** Project administration (equal). **Honghai Zhang:** Funding acquisition (equal); project administration (equal).

## CONFLICT OF INTEREST STATEMENT

No potential conflict of interest was reported by the authors.

## Supporting information


Table S1.

Table S2.

## Data Availability

All information about 16S rRNA gene data (e.g., accession numbers and DOI numbers) is listed in Table S1.
