# Correction to Comparative analysis of the gut microbiome of ungulate species from Qinghai–Xizang plateau

**DOI:** 10.1002/ece3.70356

**Published:** 2024-09-23

Wang, X., Gao, X., Chen, Y., Wu, X., Shang, Y., Zhang, Z., Zhou, S., Zhang, H. (2024). Comparative analysis of the gut microbiome of ungulate species from Qing hai–Xizang plateau. *Ecology and Evolution*, 14, e70251. http://doi.org/10.1002/ece3.70251

There are group name errors in Figure 4 and Table S2. The HA, HY, HB, and HT should be changed to EK, BG, PN, and PH, respectively. The group names have been modified in the corrected version.

We apologize for this error.

The correct Figure 4 is shown below.

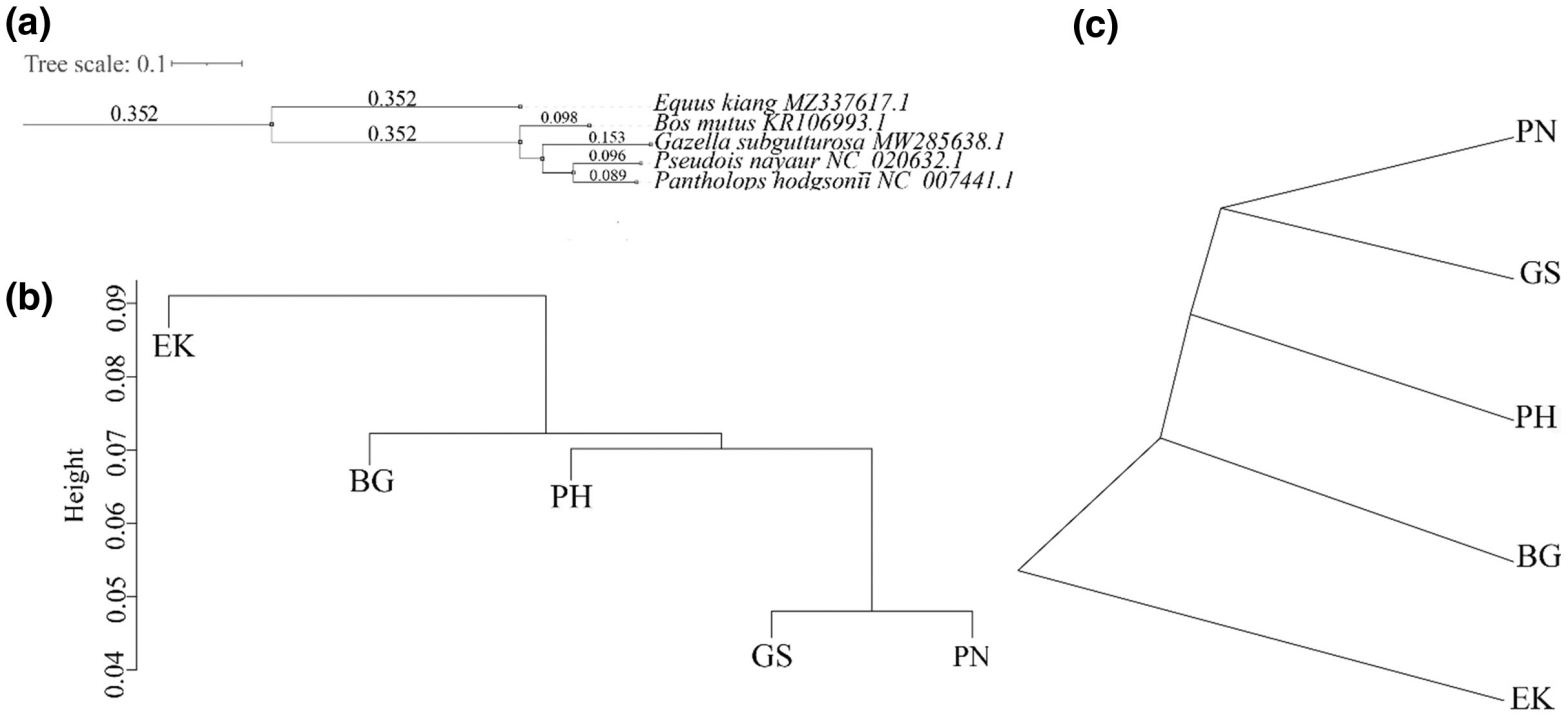

The correct sample names in Table S2 are shown below.

**Table:** 

Sample ID
EK1
EK2
EK3
EK4
EK5
EK6
EK7
EK8
EK9
EK10
PH1
PH2
PH3
PH4
PN1
PN2
PN3
PN4
BG1
BG2
BG3
BG4
BG5
BG6
BG7
GS1
GS2
GS3
GS4
GS5
GS6
GS7
GS8
GS9
GS10
GS11
GS12
GS13
GS14
GS15
GS16
GS17
GS18
GS19
GS20

